# Association mapping unveils favorable alleles for grain iron and zinc concentrations in lentil (*Lens culinaris* subsp. *culinaris*)

**DOI:** 10.1371/journal.pone.0188296

**Published:** 2017-11-21

**Authors:** Akanksha Singh, Vinay Sharma, Harsh Kumar Dikshit, Muraleedhar Aski, Harish Kumar, Nepolean Thirunavukkarasu, Basavanagouda S. Patil, Shiv Kumar, Ashutosh Sarker

**Affiliations:** 1 Division of Genetics, ICAR-Indian Agricultural Research Institute, New Delhi, India; 2 Department of Bioscience and Biotechnology, Banasthali University, Banasthali, Rajasthan, India; 3 Punjab Agriculture University, RRS, Faridkot, Punjab, India; 4 Regional Centre, ICAR- Indian Agricultural Research Institute, Dharwad, India; 5 ICARDA, B.P. 6299, Station Experiment, INRA-Quich, Rue Hafiane Cherkaoui Agdal, Rabat-Institutes, Rabat, Morocco; 6 South Asia and China Program (ICARDA), NASC Complex, New Delhi, India; National Bureau of Plant Genetic Resources, INDIA

## Abstract

Lentil is a major cool-season grain legume grown in South Asia, West Asia, and North Africa. Populations in developing countries of these regions have micronutrient deficiencies; therefore, breeding programs should focus more on improving the micronutrient content of food. In the present study, a set of 96 diverse germplasm lines were evaluated at three different locations in India to examine the variation in iron (Fe) and zinc (Zn) concentration and identify simple sequence repeat (SSR) markers that associate with the genetic variation. The genetic variation among genotypes of the association mapping (AM) panel was characterized using a genetic distance-based and a general model-based clustering method. The model-based analysis identified six subpopulations, which satisfactorily explained the genetic structure of the AM panel. AM analysis identified three SSRs (PBALC 13, PBALC 206, and GLLC 563) associated with grain Fe concentration explaining 9% to 11% of phenotypic variation and four SSRs (PBALC 353, SSR 317–1, PLC 62, and PBALC 217) were associated with grain Zn concentration explaining 14%, to 21% of phenotypic variation. These identified SSRs exhibited consistent performance across locations. These candidate SSRs can be used in marker-assisted genetic improvement for developing Fe and Zn fortified lentil varieties. Favorable alleles and promising genotypes identified in this study can be utilized for lentil biofortification.

## Introduction

Lentil (*Lens culinaris* subsp. *culinaris*) is an annual, self-pollinating, herbaceous, cool-season grain legume originating from the Near East center of origin [1]. From the Mediterranean region, the crop spread to different parts of the world, which led to the evolution of six geographical groups based on morphology, physiology, and functional variation [2]. *Pilosae*-type lentil with low biomass, small seeds, short rudimentary tendrils, pubescent foliage, and precocity in flowering and maturity is grown in South Asia. Several research groups have recommended the introgression of the Mediterranean lines [3–6] and wild species for increasing the genetic diversity and broadening the genetic base of the *pilosae*-type lentil. This crop is used mainly for food and fodder. Lentil grains are rich source of protein, fiber, minerals and carbohydrates playing a crucial role in reducing micronutrient deficiency in developing countries [7]. The crop is usually grown in rotation with cereals to break disease cycles and to fix atmospheric nitrogen [8].

The global lentil production during 2014 was 4.95 million tons [9]. Major lentil-growing regions included South Asia, West Asia, and North Africa. Micronutrient deficiency is prevalent in these regions because of high population densities and poor resources. India is a major producer and consumer of lentil. In India, lentil is grown in 1.89 million hectare with a production of 1.13 million ton [9]. This crop is mainly grown in the rain-fed areas of Central India and parts of Eastern India on residual moisture from the rainy season. The productivity of lentil is low because of short growing period and moisture stress during flowering and fruiting. Earlier study [5] has reported narrow genetic base of Indian lentil.

Micronutrients represent the essential vitamins and minerals required for normal cellular and molecular functions. Micronutrient deficiency is widespread in developing countries because of the poor quality of diet, which consists mainly of staple crops and very small amounts of meat, pulses, and fruits owing to low income. Micronutrient deficiency in food crops is primarily because of the low natural levels of available micronutrients in the soil owing to the decreased use of animal manure, crop residue and compost [10], which further results in low availability of micronutrients to plants [11]. Fe has been reported as heme and non heme forms. Non vegetarian food is source of heme iron while non heme iron is found in plants. In human beings Fe is essential constituent of many proteins and enzymes and is involved in cell growth and differentiation and oxygen transport. The heme Fe bioavailability is 12%–25% and non-heme Fe bioavailability is less than 5% [12]. Fe deficiency results in fatigue due to decreased Fe delivery as well as poor immunity and performance [13]. Fe deficiency affects more than 30% people worldwide [14]. Fe deficiency results in the disruption of the optimal function of both endocrine and immune systems [15]. It can cause anemia, which increases the risk of hemorrhage and bacterial infections during childbirth, thereby resulting in maternal deaths [16]. Babies may be born prematurely and may be liable to infections, learning disabilities, and delayed development [17]. Almost 40% pregnant women and more than 40% children under the age of 5 years in developing countries are anemic [18]. Almost 50% of these anemia cases are estimated to be due to Fe deficiency. Globally, half of the cultivated soil is zinc (Zn) deficient [19]. Zn is an essential component of proteinases, dehydrogenases, and peptidases in plants and is found in soil in the form of Zn^2+^ and Zn (OH)^+^[20]. In humans, Zn is essential for the normal growth and development of fetuses and adolescent children [21]. Regular intake of Zn is required because the human body cannot store Zn [22]. Zn deficiency impairs immune functions and is associated with an increased risk of gastrointestinal infections [23,24]. It is also a contributing factor in child deaths due to diarrhea [25].

With the emergence of PCR technology [26, 27], a new era began in molecular biology. In lentil, PCR-based markers, such as RAPD, RFLP, AFLP, inter-simple sequence repeats (ISSRs), and sequence-tagged microsatellites, were initially used for phylogenetic studies, linkage map construction, and diversity analysis within cultivated and between different *Lens* species [28–33]. Later Simple sequence repeats (SSRs) were introduced. SSRs are short tandem repeats of 1–6 bp [34]. SSRs are multiallelic, hypervariable, and chromosome specific; exhibiting codominant inheritance [35,36]. SSRs are distributed throughout the genome in both coding and noncoding regions [37]. SSRs are also present in chloroplasts [38] and mitochondria [39]. However, the development of genomic microsatellite markers is expensive, time consuming, and labor intensive and requires prior knowledge of DNA sequences [40,41]. SSRs from expressed sequence tag (EST) are associated with the transcribed regions of the genome [42,43] and can be developed through mining EST databases. Compared with genomic SSRs, EST-SSRs have a high level of transferability across related species [44–50]. Only limited reports are available on the development of genomic SSRs [30–32] and EST-SSRs [51,33] and their use in diversity analysis.

Breeding for quantitative or complex traits is a tedious and time-consuming process. Both genotypic and environmental factors play a role in the expression of a phenotype. The identification of molecular markers in the late 1980s has led to the development of tools for the directed manipulation of quantitative traits in field crops. Collard *et al*. [52] proposed a scheme for quantitative trait loci (QTL) mapping in crop plants for their further use in marker-assisted selection. Linkage analysis (using biparental populations) and association mapping (using diverse germplasm lines) are tools for dissecting quantitative traits. For linkage analysis from biparental crosses, several types of mapping populations (F2, RILs, NILs, double haploid, and backcross) can be developed. The progenies of the developed populations are screened for a trait of interest. Bulk segregant analysis, suggested by Michelmore *et al*. [53], is used to identify markers linked with the trait of interest. Although QTL mapping is a crucial tool for tagging and mapping of genes in crop plants, the method has limitations such as few meiotic events, difficulty in building a segregating population, reduced diversity derived from two parents, high cost involved, low resolution, and simultaneous evaluation of few alleles [54, 55]. The association mapping (AM) approach, also known as linkage disequilibrium (LD) mapping [56, 57], has been initially used by geneticists to map and clone many genes governing complex traits in humans [58–60]. However, in recent years, plant geneticists have also used it for identifying QTL in different crops [61–67]. AM harnesses genetic diversity of natural population, for searching genotype-phenotype correlations among unrelated individuals. The main advantage of AM is likelihood for a higher resolution mapping because of the utilization of majority recombination events from a large number of meiosis throughout the germplasm development history [68]. The association mapping approach is based on the LD between the marker and the QTL, and it provides higher mapping resolution. The detection power of QTL depends on the LD between the QTL and the marker. A strong LD between the markers enhances the detection of QTL with a small effect [69]. The variance explained by QTL is underestimated if the LD between the marker and QTL is incomplete. In this study, we used association mapping for identifying QTL linked to grain Fe and Zn concentration in lentil. Inductively coupled plasma-mass spectrometry (ICP-MS) was used to estimate grain Fe and Zn concentration in a set of 96 Indian and Mediterranean lentil genotypes with 73 genomic and EST-SSRs. We then conducted an association mapping study by using a general linear model (GLM) to detect the significant loci responsible for natural variations of grain concentrations of Fe and Zn. We present results on significantly associated loci and favorable alleles. This approach can be used for identifying germplasm lines with desirable traits for accelerating lentil breeding through marker-assisted selection.

## Material and methods

### Plant material and field experiment

An AM panel of 96 diverse *L*. *culinaris* subspecies *culinaris* genotypes was used in this study (Table 1). The panel consisted of advanced breeding lines developed at different lentil breeding centers of India, released lentil varieties, and exotic germplasm lines obtained from the International Center for Agricultural Research in the Dry Areas (ICARDA). The genotypes were selected (based on origin and seed size) from global lentil germplasm maintained at Indian Agricultural Research Institute, New Delhi. The panel included both microsperma (small seeded) and macrosperma (large seeded) lentil types. The selected genotypes exhibited highly significant variation for both grain Fe and Zn concentration (S1 and S2 Tables) and almost normal distribution for these traits (S1 Fig). The genotypes of panel were grown at three geographical locations: (i) Delhi (North-West Plain Zone; 28°40′N 77°12′E, 218 meters above the sea level [masl]), during 2013–14 and 2014–15 (ii) Indore (Central Zone; 30.9°N 75.85°E, 244 masl, during 2013–14 and (iii) Dharwad (Peninsular Zone; 28°58′N 79°25′E 344 masl) during 2013–14. The soil characteristics (pH, EC, organic carbon content, available N, P and K and soil texture) of Delhi, Dharwad and Indore are presented in S3 Table. In previous season mungbean was planted across all the three locations in these plots. No micronutrient spray or basal application was given. Only fertilizer applied to mungbean crop was 100 kg Di ammonium Phosphate / ha. To ensure proper homogenization, the soil was pulverized and thoroughly mixed and the field was leveled at each location. The experiment was planted in two replication. From each replication in each location ten samples were drawn to estimate soil Fe and Zn reveal significant variation ensuring the plot homogeneity. The panel was planted in a randomized complete block design with two replicates per entry (3 rows per replication) with a plant distance of 5 × 30 cm and a row length of 4 m. Standard agronomic practices were followed for crop cultivation. Fe and Zn concentration in the soil were estimated using a procedure proposed by Singh *et al*. [70].

**Table 1:** Source / Origin of 96 genotypes of lentil used in the study.

S.No.	Genotypes	Source	S.No.	Genotypes	Source
1	L 404	IARI, New Delhi	49	L 4590	IARI, New Delhi
2	L 830	IARI, New Delhi	50	PL 4	GBPUAT, Pantnagar
3	L 4596	IARI, New Delhi	51	L 4591	IARI, New Delhi
4	L 4602	IARI, New Delhi	52	LL 1203	PAU, Ludhiana
5	L 4603	IARI, New Delhi	53	RLG 147	ARS, Durgapura
6	L 4618	IARI, New Delhi	54	DL 11–4	TCA, Dholi
7	L 4620	IARI, New Delhi	55	KLS 113	CSAU, Kanpur
8	L 4648	IARI, New Delhi	56	NDL 11–1	NDUAT, Faizabad
9	L 4649	IARI, New Delhi	57	PL 122	GBPUAT, Pantnagar
10	L 4650	IARI, New Delhi	58	SKUAL 9	Srinagar
11	L 4698	IARI, New Delhi	59	L 4706	IARI, New Delhi
12	L 5120	IARI, New Delhi	60	DPL 15	IIPR, Kanpur
13	L 5126	IARI, New Delhi	61	LL 1210	PAU, Ludhiana
14	L 5253	IARI, New Delhi	62	KLB 345	CSAU, Kanpur
15	ILL 7663	ICARDA, Aleppo, Syria	63	PL 024	GBPUAT, Pantnagar
16	L 7818	IARI, New Delhi	64	PL 129	GBPUAT, Pantnagar
17	L 7903	IARI, New Delhi	65	IPL 324	IIPR, Kanpur
18	DPL 15	IIPR, Kanpur	66	IPL 406	IIPR, Kanpur
19	DPL 21	IIPR, Kanpur	67	L 4707	IARI, New Delhi
20	DPL 58	IIPR, Kanpur	68	LL 1204	PAU, Ludhiana
21	PL 02	GBPUAT, Pantnagar	69	LH 84–8	HAU, Hisar
22	P 13129	ICARDA, Aleppo, Syria	70	RVL 48	Sehore
23	PL 101	GBPUAT, Pantnagar	71	KLB 314	CSAU, Kanpur
24	PL 639	GBPUAT, Pantnagar	72	IPL 325	IIPR, Kanpur
25	RL 1	IARI, New Delhi	73	DPL 62	IIPR, Kanpur
26	ILL 2581	ICARDA, Aleppo, Syria	74	P 2102	ICARDA, Aleppo, Syria
27	SKL 259	IARI, New Delhi	75	P 2124	ICARDA, Aleppo, Syria
28	EC 1	IARI, New Delhi	76	P 2125	ICARDA, Aleppo, Syria
29	10-2-B-2	IARI, New Delhi	77	P 2126	ICARDA, Aleppo, Syria
30	10-3-Y-26	IARI, New Delhi	78	P 2127	ICARDA, Aleppo, Syria
31	Globe mutant	IARI, New Delhi	79	P 2130	ICARDA, Aleppo, Syria
32	Fasciated mutant	IARI, New Delhi	80	P 2205	ICARDA, Aleppo, Syria
33	HM 1	HAU, Hisar	81	P 2215	ICARDA, Aleppo, Syria
34	MC 6	IARI, New Delhi	82	P 2230	ICARDA, Aleppo, Syria
35	K 75	CSAU, Kanpur	83	P 2233	ICARDA, Aleppo, Syria
36	VL 103	VPKAS, Almora	84	P 2239	ICARDA, Aleppo, Syria
37	FLIP 96–57	ICARDA, Aleppo, Syria	85	P 3113	ICARDA, Aleppo, Syria
38	LL 147	PAU, Ludhiana	86	P 3204	ICARDA, Aleppo, Syria
39	LL 931	PAU, Ludhiana	87	P 3208	ICARDA, Aleppo, Syria
40	LC 74-1-5-1	IARI, New Delhi	88	P 3220	ICARDA, Aleppo, Syria
41	LC 300–1	IARI, New Delhi	89	P 13104	ICARDA, Aleppo, Syria
42	PL 4	GBPUAT, Pantnagar	90	P 13113	ICARDA, Aleppo, Syria
43	LL 1231	PAU, Ludhiana	91	P 13122	ICARDA, Aleppo, Syria
44	IPL 221	IIPR, Kanpur	92	P 13135	ICARDA, Aleppo, Syria
45	VL 143	VPKAS, Almora	93	P 13143	ICARDA, Aleppo, Syria
46	PL 406	GBPUAT, Pantnagar	94	P 14103	ICARDA, Aleppo, Syria
47	PL 117	GBPUAT, Pantnagar	95	P 14201	ICARDA, Aleppo, Syria
48	IPL 220	IIPR, Kanpur	96	P 15104	ICARDA, Aleppo, Syria

### Analysis of grain sample for Fe and Zn concentration

The grains of each genotype were harvested separately at maturity. Care was taken to prevent contamination from dust and metallic equipment. Plants were hand-threshed to avoid any type of contamination. Then the grains were placed in a clean plastic tray manually (using contaminant-free gloves). The grains of each sample were washed with Milli-Q water and dried at 35°C for five days in a contamination-free uncorroded oven. From each sample, 10 g of grains was grounded manually into a fine powder using a mortar and pestle. The micronutrient analyses were performed at the Division of Soil Science and Agricultural Chemistry, IARI, New Delhi, India. The grain powder sample (0.5 g) was digested following the modified diacid protocol [70] by using a microwave digestion system (Multiwave ECO, Anton Paar, les Ulis, France). Fe and Zn concentration (in mg/kg seed) were estimated through ICP-MS (Perkin Elmer, model: NexION 300 ICP-MS, USA) by using an automatic sampling protocol. Two technical replications per biological replication were followed while estimating the concentration.

### Genomic DNA extraction, purification and SSR amplification

Genomic DNA of the genotypes was extracted from 5 g of fresh leaf tissue by using the CTAB method proposed by Murray and Thompson [71]. The quality and quantity of DNA were determined using a spectrophotometer, and the samples were diluted to 10 ng/μL. The PCR mixture (20 μL) consisted of a 10X buffer (100 mM Tris-HCl, 15 mM MgCl_2_, and 500 mM KCl), 0.5 μM each of forward and reverse primers, 200 μM of each dNTP, 1 U of Taq DNA polymerase, PCR reagents, an EST-SSR or genomic SSR primer procured from Sigma-Aldrich (Spruce Street, St. Louis, USA) and approximately 40 ng of template genomic DNA. PCR was performed in a VeritiTM thermal cycler (Applied Biosystems, Life Technologies, Singapore) using the following temperature cycle: one denaturation cycle at 94°C for 4 min, followed by 30 cycles of 94°C for 1 min, annealing at 59°C–62°C (primer specific) for 30 sec, extension at 72°C for 1 min, and a final extension at 72°C for 10 min. The amplified products were electrophoresed for 3 h on 3% metaphor TM agarose gels (Lonza, Rockland, ME USA) at a constant voltage of 100 V in 1X TBE buffer. The gel was stained using ethidium bromide and visualized. The amplicons were photographed under UV light with a CCD camera attached to a gel documentation system (Alpha Imager) at 260 nm. A 50-bp DNA ladder (MBI, Fermentas, Vilnius, Lithuania) was used as a molecular size marker. Sixty genomic SSRs and 260 EST-SSRs were used to study polymorphism. Of the 73 polymorphic markers identified for genotyping, 20 were genomic SSRs [72,30, 31] and 53 were EST-SSRs [33, 51].

### Diversity analysis and population structure

Polymorphism information content (PIC) was computed using the formula PIC = 1-ΣPi − ΣΣPi Pj, where “i” is the total number of alleles detected for the SSR marker, “Pi” is the frequency of the i allele in the set of 96 genotypes investigated, and j = I + 1 [73]. A binary matrix was then transformed to a genetic similarity matrix by using Jaccard’s coefficient. Unweighted neighbor joining (UNJ) method available in DARwin 5.0 (http://darwin.cirad.fr/) was used to visualize the dendrogram.

STRUCTURE 2.3.4 was used [74] to determine the number of subgroups in the population assuming prior values of k = 1 to 10. The data was analyzed at a run length of 2,50,000 as the burning period length followed by 2,50,000 Markov Chain Monte Carlo iterations by keeping α constant. Each k value was repeated 10 times with an admixture model and correlated the allele frequency. The optimum k value was determined by plotting the Ln P (D) value against the given k value. Structure harvester v 6.92 [75] was used for obtaining the optimum k value determined by plotting the Ln P (D) value against k. The highest plateau was observed at delta k = 6; hence, the number of inferred populations was assumed to be six for further analysis.

### Association mapping and favorable allele mining

To identify QTL for grain Fe and Zn concentration, association analysis were performed using 73 SSRs. The LD was estimated between each pair of polymorphic loci by calculating the square of the correlation coefficient (r^2^) using TASSEL 3.01 [76] with 10,000 permutation. The General Linear Model (GLM with Q matrix to reduce false associations) was used to examine association between grain micronutrients and markers. An LD plot with p and r^2^ values was generated by TASSEL to depict the overall LD among the entire SSR set. The markers considered to be significantly associated with the trait are represented in Manhattan plot [76]. Quantile-quantile (QQ) plots of the expected and observed p values was plotted to evaluate the adequacy of controlling Type I error.

Favorable allele for a marker loci associated with grain Fe and Zn concentration were identified using formula suggested by Li *et al*. [77]. The phenotypic allele effect (ai) was calculated as a_i_ = ∑x_ij_/n_i_ − ∑N_k_/n_k_, where a_i_ is the phenotypic effect of the i_th_ allele, x_ij_ is the phenotypic value over the j_th_ material with the i_th_ allele, n_i_ is the number of genotypes with the i_th_ allele, N_k_ is the phenotypic value over all genotypes, and n_k_ is the number of genotypes [78]. It represents a comparison of the average phenotypic value of genotypes with a specific allele with that of all genotypes. When, *ai* > 0, then this allele is supposed to have positive effect on the trait. When *ai* < 0, the allele gives a negative effect [79].

## Results

### Phenotypic variation in lentil germplasm

The variation in grain Fe and Zn concentration across locations and years are shown in the S1 Table. The average soil Fe and Zn concentration were 5.01 mg/kg and 1.68 mg/kg at Delhi (2013–14), 5.23 mg/kg and 1.62 mg/kg at Delhi (2014–15), 4.2 mg/kg and 0.62 mg/kg at Indore (2013–14) and 4.5 mg/kg and 0.985 mg/kg at Dharwad (2013–14), respectively. The mean grain Fe concentration ranged from 31.55 to 119.35 mg/kg and that of Zn ranged from 7.80 to 75.45 mg/kg.

In this study, efforts were made to identify lentil genotypes with high grain Fe and Zn concentration based on the multilocation data. The range for Fe concentration at Delhi was 38.4–119.35 mg/kg during 2013–2014 and 34.4–115.35 mg/kg during 2014–2015. Indore and Dharwad exhibited a range of 40.52–111.4 mg/kg and 31.55–106.05 mg/kg, respectively, during 2013–2014. Considering the performance of the studied genotypes in all the environments, L 4596 (122.46 mg/kg) was the most promising genotype, followed by L 5126 (114.96 mg/kg), whereas P 3226 had the lowest grain Fe concentration (27.52 mg/kg). The range for Zn concentration at Delhi was 12.3–74.15 mg/kg during 2013–2014 and 12.65–78.75 mg/kg during 2014–2015. Indore and Dharwad exhibited a range of 7.4–63.65 mg/kg and 22.25–62.95, respectively. Considering the data from the four environments, P 3220 (78.75 mg/kg) and P 13129 (70.45 mg/kg) were determined as the most promising genotype for the grain Zn concentration. The indigenous lines were found to be rich in grain Fe, whereas the exotic lines were found to be rich in grain Zn. A mean Fe grain concentration higher than 70 mg/kg seed was considered high, whereas that lower than 60 mg/kg seed was considered low. Similarly, a mean Zn grain concentration higher than 50 mg/kg seed was considered high and that lower than 40 mg/kg seed was considered low. Promising genotypes such as P 2130 (Fe: 85.75 mg/kg; Zn: 61.27 mg/kg), P 2126 (Fe: 102.26 mg/kg; Zn: 62.74 mg/kg), and L 4596 (Fe: 116.19 mg/kg; Zn: 47.38 mg/kg) were found for both grain Fe and Zn. The genotypic and phenotypic correlation matrix for grain Fe and Zn concentration for different geographical regions and years is presented in S2 Table. A significant genetic correlation was recorded for the grain Fe concentration among different geographical regions during 2013–2014. Similar results have been recorded for the grain Zn concentration. A low correlation was recorded between grain Fe and Zn concentration among different geographical locations and years. A histogram depicting the distribution of genotypes for grain Fe and Zn concentration is shown in S1 Fig.

### Genetic diversity and population structure

A total of 220 alleles were generated through 73 polymorphic SSRs in the AM panel (Table 2). The number of alleles produced per locus ranged from 2 to 5, with an average of 2.97 alleles per locus. The highest number of alleles (5) was detected for SSRs PBALC 353 and SSR 33, whereas 17 SSRs exhibited only two alleles. The PIC value ranged from 0.08 to 0.68, with an average of 0.36, indicating that the SSRs used in the study were informative. Genomic SSRs produced a higher average number of alleles (Na), and PIC over EST SSRs (Table 2). SSR 33 produced the maximum number of alleles (5 with the highest PIC value of 0.68).

**Table 2:** List of the 73 EST and genomic SSRs primers used in present study.

S.No	Primer	Primer sequence	T_a_°C	Na	PIC
1	PLC5	F:CATTGCAGCTTATTCTCACAGC R:TGACCCATCCTCATCCTTAAAT	60	4	0.63
2	PLC10	F:TGCAACAAAGGACACTAGAGGTT R:ATTTCTTTCTCCCTAACCAGCC	59	3	0.3
3	PLC16	F:CGTTTGATCTTCTAAGCCCCTA R:AAGGGAAAGGATGTTTGACTTG	59	3	0.41
4	PLC17	F:AAGCTGAAGGAAATCAAAGTGG R:TCAACACACTCCATGTTTAGAGC	59	3	0.44
5	PLC21	F:AACTCGCATCCTCTTCACAACT R:GGACCTTTCCCTTGTAGTCACC	58	3	0.24
6	PLC30	F:TTGGTCAGGTTCTCAATCCTCT R:ACGGATGAACGCTTGTAAAGAA	61	3	0.47
7	PLC35	F:TTGCTTCCTCCTCTTCTCACTC R:AGCCTCAGTACCCTCCTCTTTT	60	3	0.36
8	PLC38	F:CCTGGAGAAGTCTGTGGAAGAT R:AGCTCTAGCATTTTGCATGTGA	59	2	0.36
9	PLC39	F:CAGAGAAATCCCCTGCTGAG R:CATGATTCCCATAGCCTTGC	58	3	0.29
10	PLC40	F:CAACTCGCATCCTCTTCACA R:CAAAGGGGTTGGAGTCGTAA	60	4	0.43
11	PLC42	F:AACCAATCATGGCTTCTGCT R:TTTCACCGTCTTTATGAACCA	60	4	0.66
12	PLC46	F:CAAACTGGAAGATGCTGCTG R:TGACCCATCCTCATCCTTAAA	60	3	0.22
13	PLC51	F:CCATGATGAGCCTTGAATGA R:TCTTCAATCTCCAGGAACACTTT	62	4	0.52
14	PLC60	F:TGCTTGGACCCTAAATTTGC R:AAGAAAAGGGCAACCACTGA	60	3	0.29
15	PLC62	F:AAGCCAACCATTTTTGCATC R:AGTAATCCTTTGGTGCTGCG	58	4	0.53
16	PLC63	F:TTGATGGCTATGGGAGTGGT R:TGGTCCCAACAAAATACCAA	60	3	0.19
17	PLC74	F:GATTTACCGATGGATCTTCA R:CTAAGGGAGAGAAAGAAAAGG	61	2	0.08
18	PLC77	F:GGAAAGAGCCAAGAAGTTG R:ACCCATCCTCATCCTTAAAT	56	3	0.49
19	PLC80	F:GCTAACAAACAACACCATGA R:GCATCTAAGTTCTTCAATCTCC	58	3	0.25
20	PLC81	F:GGGTAGAGTATTATTGAAGGTGG R:AGAATCGCTAGTTTAGAGCAAG	60	3	0.44
21	PLC83	F:GTTCGGTTTTGTTGGAAGTGAG R: TCCTTCTTTCAGCCATGAGATT	60	3	0.35
22	PLC88	F:CCAAAACAAGCACCAGTACAAG R:TAGAAGACGTTGGAGGAGAAGC	59	3	0.42
23	PLC95	F:TTCATTCTTGGGCTAGGGA R:TGCAGATGTGAAATACCTCAGT	59	2	0.21
24	PLC96	F:TTCATCGTCGTTAATCGGAAC R:GAGAGGAAGGACATTGGAAGAA	59	2	0.22
25	PLC98	F:GTGCGGTGTTGTTGTATTGATT R:TCTTTAGCTTCTTCCAAAACGG	59	3	0.51
26	PLC100	F:TGCTTTACTTCCTTCTCTCTTTGC R:TAAGCCATCCACTTGCATCC	60	3	0.51
27	PLC104	F:AGCTGTTGATTTTGGCGG R:CCGCAGATCCAGAAAAGAAG	59	3	0.54
28	PBALC2	F:GATGCGACGCAGAAGATTAAG R:TGACCATAACCATTCCTCTGAT	59	2	0.24
29	PBALC6	F:ATGATCCGAGTTTCCTGCA R:TACACCACCAACTTCCACCA	60	3	0.24
30	PBALC13	F:GCAGCAGCATGAGAAAATG R:ATTACTCGACGCCCCCTAGT	60	3	0.33
31	PBALC18	F:CGTTGGTGGTGCAGTATTTG R:CCATAAACAAGTGCAATCCAG	60	2	0.2
32	PBALC25	F:ACCCCTGCAAATGTCAAGAG R:AAATCCAAATGCATAACTTCATTG	60	2	0.15
33	PBALC29	F:TATGCCATTGGATGTGGTTG R:TATTCAGTTTCCGCCAAAGG	60	3	0.22
34	PBALC32	F:CTGGAGGGAAAAGATGACGA R:TTTCCCCAACTTTCCTAAGC	60	4	0.45
35	PBALC43	F:GCATGGTTAAGAAGAAGGGTGT R:TAACAACAAACAAGCGCATTA	61	3	0.33
36	PBALC203	F:CATAGTCAACACTTGGTCGTT R:GTCCACAATGAAACTCATCAC	60	3	0.63
37	PBALC205	F:TTGAGTTTGAGGATGAGGATA R:CATAAAACCCCAAACATTACA	59	2	0.11
38	PBALC206	F:GATCCTGTTTTATCCCATTGT R:ACAATCACTTAGCCAAAATCA	59	2	0.29
39	PBALC207	F:ATGGAACACAAACCAATACAC R:TGTGGTGTCCTTTGTAGAAGT	59	2	0.44
40	PBALC213	F:AAGTTTGGGATAAACCTTTTG R:CATCATGCTAAAATCAAAACC	61	3	0.31
41	PBALC216	F:AAATAGAAGTGGAGAGGCAAT R:TTCGTTCTTGAGTGATATCGT	60	3	0.33
42	PBALC217	F:TTACCAAGAAATTGAATACAGC R:AGTTTGAAAGGATCTCCAAAG	60	3	0.17
43	PBALC219	F:TAGCAAATGGACGTGTAGAGT R:GTGGTGCTCAATACACAATCT	60	2	0.26
44	PBALC224	F:CCACCCACTTACAAGTACAAA R:TAAATTGGTGGTGGTGAGTAA	59	2	0.15
45	PBALC238	F:CGCAATCCAAACCTAATCTAT R:TTCTAGGATGTGATTTTGGTG	59	2	0.36
46	PBALC250	F:TGCATTTACCATCATCTCTAAC R:TGATTGATTCGGTACTTTTTG	60	3	0.48
47	PBALC254	F:ATGTTAATAAGCAGCAGCAAC R:AAGTTGCATGTAACCACAAAC	60	4	0.32
48	PBALC260	F:GTGAACTACCTCTGTGAATGC R:AGGCGAAATTTCATCTTCTA	60	4	0.4
49	PBALC265	F:AACATAAAGGAGAGGGTCATC R:CATCTTGTCAACAATTCCTTC	59	4	0.38
50	PBALC347	F:CAAAAATGGCTACTTTGATTG R:GCTTCAGATCAACTGTCTCAG	59	3	0.31
51	PBALC353	F:CCATAACAGACAAAACCCTACT R:ATTCTCAAAGCCCATTTAGTT	59	5	0.34
52	PBALC742	F:AGCAAATTCTATTCCAACACA R:CCAATTCTACTTCCACCTTCT	59	2	0.49
53	PBALC762	F:TGATGGAACCAAACTTCTTTA R:TATCCTCCCTAAAATCAAAGG	59	3	0.31
Mean				2.94	0.35
54	GLLC106	F:ACGACAATCCTCCACCTGAC R:AACAAGGAAGGGGAGAGGAG	56	3	0.18
55	GLLC108	F:CGACAATCCTCCACCTGAC R:ACAAGGAAGGGGAGAGGAAG	56	4	0.27
56	GLLC527	F:GTGGGACGGTTTGAATTTGA R:GAACATAAAATGGGAGTGTCACAA	56	3	0.44
57	GLLC538	F:AAGGGAAGGAAAAGGGAAGT R:GCACGAAGAGGGTACGTAGG	56	2	0.23
58	GLLC541	F:TGGGCTCATTGAACCAAAAG R:CCCCCTTTTAAGTGATTTTCC	56	3	0.47
59	GLLC562	F:TGTGTAGGCACATCAACAAAA R:GGTGGGCATGAGAGGTGTTA	55	4	0.47
60	GLLC563	F:ATGGGCTCATTGAACAAAAG R:CCCCCTCTAAGAGATTTTCCTC	56	3	0.59
61	GLLC598	F:TGGGCTCATTGAACCAAAAG R:CCCCCTTCTAAGTGATTTTCC	55	3	0.27
62	GLLC609	F:GCGACATGGAATTGGATTTG R:GCACAAAGTCGAGGAGCCTA	55	3	0.58
63	GLLC614	F:AACCCCAGCCAGATCTTACA R:AAGGGTGGTTTTGGTCCTATG	55	3	0.54
64	SSR132RN	F:CCAGAACAAACGTAAACC R:CTATCGCATATGAGTGAAC	52	3	0.1
65	SSR230	F:CCAACAACAATTCACCATAC R:AACATTGTACTGAGAGGTG	53	3	0.51
66	SSR317-1	F:GTGGGTGTAATTATTGCTAC R:GTATCAAACTTATGGTGAAATC	53	3	0.57
67	SSR66	F:GGTAGTGGTGAGGAATGAC R:GCATCACTGCAACAGACC	55	3	0.4
68	SSR72	F:CAAACAGTACAAGGAAAGGAG R:CTGACTGAGCTGCTTGAAC	55	2	0.29
69	SSR302	F:CAAGCCACCCATACACC R:GGGCATTAAGTGTGCTGG	56	3	0.19
70	SSR309-2	F:GTATGTCGTTAACTGTCGTG R:GAGGAAGGAAGTATTCGTC	50	2	0.15
71	SSR48	F:CATGGTGGAATAGTGATGGC R:CTCCATACACCACTCATTCAC	57	3	0.55
72	SSR33	F:CAAGCATGACGCCTATGAAG R:CTTTCACTCACTCAACTCTC	56	5	0.68
73	SSR233	F:CTTGGAGCTGTTGGTC R:GCCGCCTACATTATGG	52	3	0.43
Mean				3.05	0.39

Ta = Annealing temperature, PIC = Polymorphism information content, Na = number of alleles

**Fig 1:**
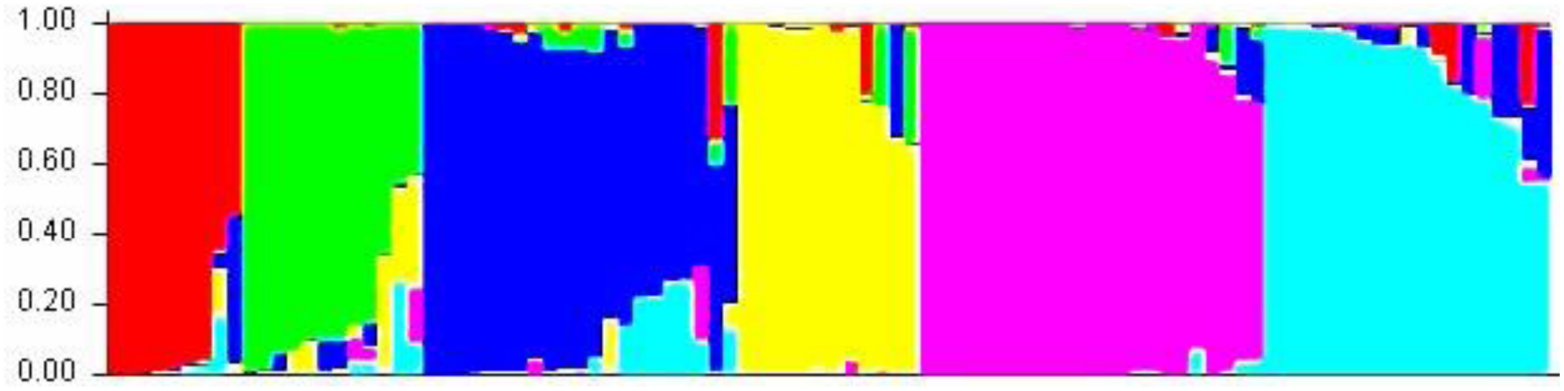
STRUCTURE plot of the lentil association mapping population with K = 6 clusters based on all polymorphic SSRs.

Admixture model-based simulations revealed six subgroups (SGs) in the panel. The six SGs represented as SG I, SG II, SG III, SG IV, SG V, and SG VI, which included 9, 12, 21, 12, 23, and 19 genotypes, respectively (Fig 1). SG I comprised of nine indigenous genotypes including released varieties and advanced breeding lines. SG II comprised of twelve genotypes including two exotic genotypes. SG III consisted of 21 genotypes including two exotic genotypes. SG IV comprised 12 indigenous genotypes developed at IARI, New Delhi. SG V included 23 exotic genotypes from ICARDA. SG VI comprised 19 indigenous genotypes. The grouping of genotypes in SG I, IV, V and VI was based on the origin of genotype. The SG II and III included mainly indigenous genotypes and few exotic genotypes.

**Fig 2:**
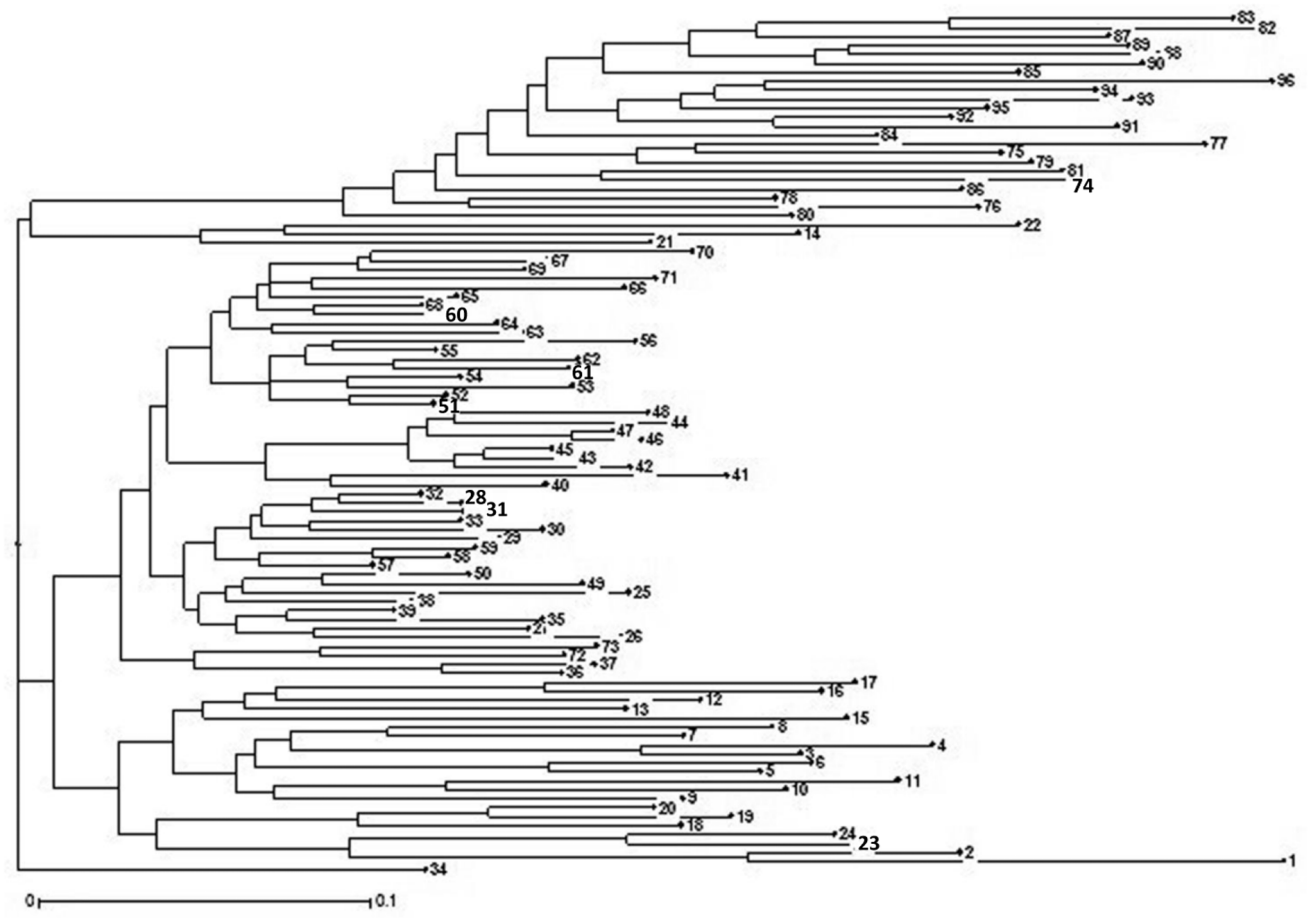
Neighbour joining dendrogram of 96 genotype of lentil with 73 SSRs (Serial number of genotype in the figure corresponds with serial number and genotype in Table 1).

The UNJ separated the lentil genotypes into three clusters. Cluster I comprised 25 lentil genotypes including 23 exotic germplasm from ICARDA and two Indian lentil genotypes L 5253 and PL 02. Cluster II comprised 70 indigenous genotypes including advanced breeding lines and released varieties including breeding material from India. Cluster III comprised only one Indian genotype, namely MC 6. The genotypes within clusters I and II are further grouped into smaller subgroups on the basis of their origin and types. Most of the exotic genotypes from ICARDA were grouped in the upper branches of the dendrogram, whereas the advanced breeding lines developed at different lentil breeding centers of India, released lentil varieties, were grouped in lower branches (Fig 2).

### LD and marker trait association analysis

**Fig 3:**
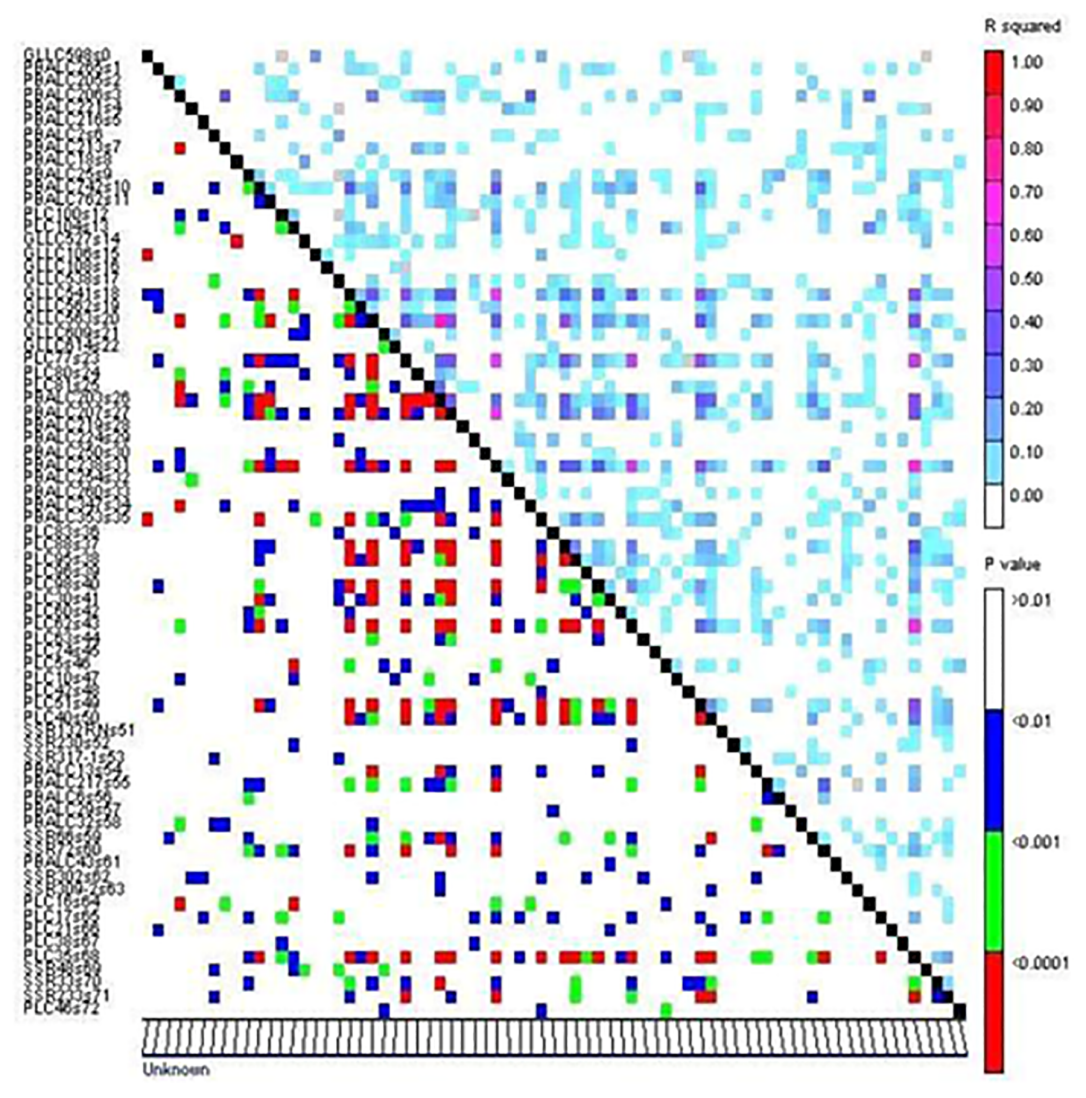
Linkage disequilibrium patterns among 96 genotypes genotyped with 73 SSRs. The squared correlation coefficients (r^2^) for each pair of markers are presented in the upper triangle and their corresponding p values in the lower triangle.

**Fig 4:**
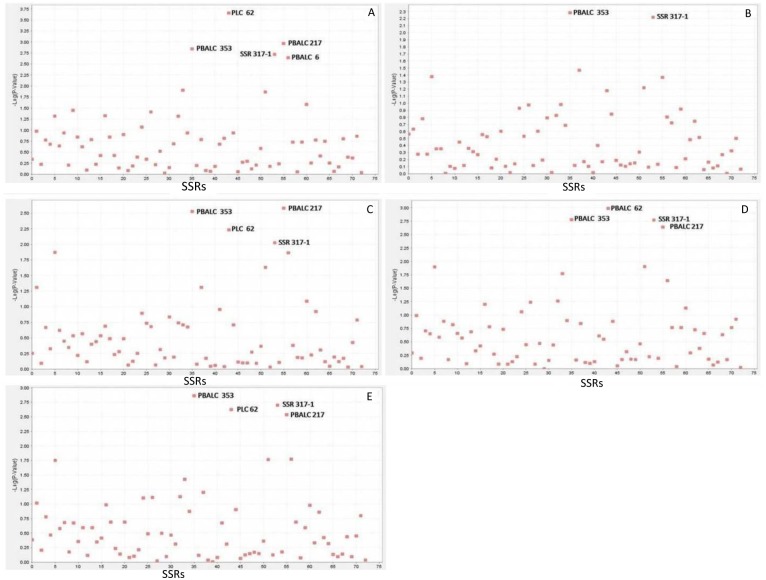
Manhattan plot depicting association of 73 SSRs markers with grain iron concentration for (A) Delhi (2013–14) (B) Dharwad (2013–14) (C) Indore (2013–14) (D) Delhi (2014–15) (E) Combined all location year.

**Fig 5:**
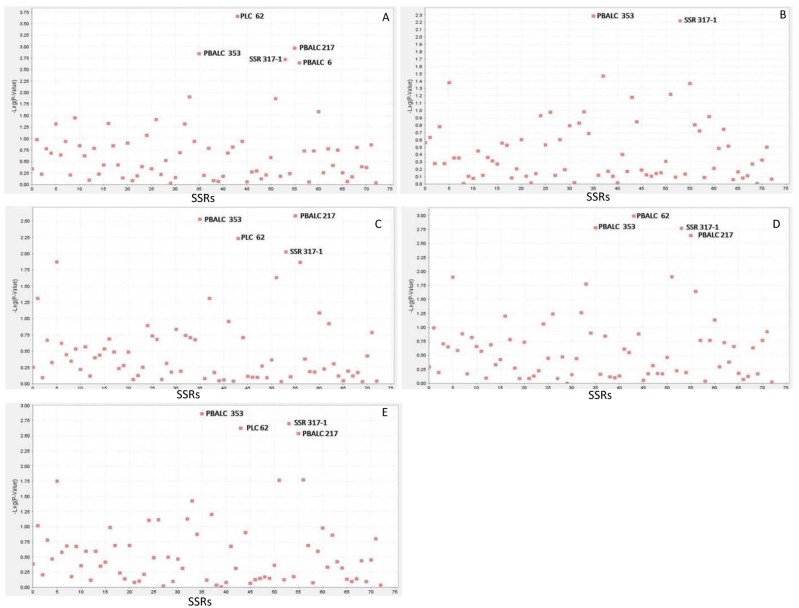
Manhattan plot depicting association of 73 SSRs markers with grain zinc concentration for (A) Delhi (2013–14) (B) Dharwad (2013–14) (C) Indore (2013–14) (D) Delhi (2014–15) (E) Combined all location years.

The LD patterns of all 2628 pairwise combinations of the 73 SSRs were assessed using TASSEL (Fig 3). The LD ranged from 0.0 to 0.70. The highest LD value was recorded between PLC 81 and GLLC 563, PLC 38, and PLC 60. In the present study, association mapping was used to identify linked markers for grain Fe and Zn concentration by using the GLM with the Q model. In total, eight SSRs (contributing to 8–22% of the phenotypic variation) were significantly associated with the grain Fe concentration, and five SSRs (contributing to 4–21% of the phenotypic variation) with the grain Zn concentration (Table 3). Environment-wise different SSRs associated with grain Fe and Zn concentration with -log10 p value >2 are presented in Figs 4 and 5. For the grain Fe concentration, the marker PBALC 13 was consistently identified in all four datasets; GLLC 563 in three datasets (Indore 2013–14, Delhi 2014–15 and Delhi 2013–14) and PBALC 206 (Dharwad 2013–14 and Delhi 2014–15) and PBALC 32 (Delhi 2013–14 and Delhi 2014–15) in two datasets each. For the grain Zn concentration, the marker PBALC 353 was consistently identified in all four datasets and SSR 317–1 (Delhi 2013–14, Delhi 2014–15, and Dharwad 2013–14), PLC 62 (Delhi 2013–14, Delhi 2014–15 and Indore 2013–14) and PBALC 217 (Delhi 2013–14, Delhi 2014–15 and Indore 2013–14) in three datasets each. Few SSRs exhibited a consistent association with grain Fe and Zn concentration across environments. Three SSRs (PBALC 13, PBALC 206, and GLLC 563) exhibiting an association with the grain Fe concentration revealed phenotypic variation of 11%, 9%, and 11%, respectively. SSRs PBALC 353, SSR 317–1, PLC 62, and PBALC 217 were found to be associated with the grain Zn concentration with the phenotypic variation of 21%, 18%, 14%, and 16%, respectively. The markers that were significantly associated with the trait were represented in Manhattan plots (Figs 4 and 5). A QQ plot is a graphical method of depicting the observed and expected probability distributions by plotting their quantiles next to each other. The results derived from the GLM analysis were also explained in these plots (S2 Fig). The hypothetical QQ plots of the marker-trait association study for grain Fe and Zn concentration are a good approximation of normality.

**Table 3:** List of significantly associated marker with grain Fe and Zn concentration.

Trait	SSR marker	Location	Year	P value	r^2^ value
Iron	PBALC13	Dharwad	2013–14	0.0030	0.0897
		Delhi	2014–15	0.0021	0.0963
		Indore	2013–14	0.0006	0.116
		Delhi	2013–14	0.0021	0.0963
		Combined all location years		0.0006	0.1168
	PBALC 221	Dharwad	2013–14	0.0053	0.1065
	PBALC 206	Dharwad	2013–14	0.0063	0.0767
		Delhi	2014–15	0.0011	0.1073
		Combined all location years		0.0019	0.09719
	PBALC 32	Delhi	2014–15	0.0047	0.1505
		Delhi	2013–14	0.0047	0.1505
	GLLC 563	Delhi	2013–14	0.0048	0.1084
		Indore	2013–14	0.0012	0.1354
		Delhi	2014–15	0.0048	0.1084
		Combined all location years		0.0043	0.1104
	PBALC 265	Indore	2013–14	0.0031	0.2276
	PBALC 207	Indore	2013–14	0.0039	0.0852
	PBALC 203	Indore	2013–14	0.0054	0.1061
	PBALC 265	Indore	2013–14	0.0031	0.2276
Zinc	PBALC 353	Dharwad	2013–14	0.0052	0.1835
		Indore	2013–14	0.0030	0.1960
		Delhi	2014–15	0.0014	0.2115
		Delhi	2013–14	0.0017	0.2081
		Combined all location years		0.0013	0.2121
	SSR 317–1	Dharwad	2013–14	0.0061	0.1631
		Delhi	2014–15	0.0019	0.1877
		Delhi	2013–14	0.0017	0.1901
		Combined all location years		0.0020	0.1868
	PLC 62	Delhi	2014–15	0.0002	0.190
		Indore	2013–14	0.0059	0.1263
		Delhi	2013–14	0.0010	0.1606
		Combined all location years		0.0024	0.1443
	PBALC 217	Delhi	2014–15	0.0011	0.1803
		Indore	2013–14	0.0026	0.1624
		Delhi	2013–14	0.0023	0.1652
		Combined all location years		0.0029	0.1605
	PBALC 6	Delhi	2014–15	0.0022	0.123

### Favorable allele mining

Phenotypic allele effects (ai) were calculated for SSRs associated with grain Fe and Zn concentration. The a_i_ value greater than zero indicates that the allele has a positive effect, whereas the value of a_i_ less than zero indicates that the allele has a negative effect. The favorable alleles identified for the grain Fe concentration include PBALC 13–1, PBALC 206–1, GLLC 563–1, GLLC 563–2, and PBALC 32–1, of which PBALC 13–1 exerted the maximum positive effect on a phenotype. Similarly, the favorable alleles identified for the grain Zn concentration include PBALC 353–3, PLC 62–3, and PLC 62–4. PLC 62–4 had the maximum positive effect on the grain Zn concentration. The representative genotypes of favorable alleles for grain Fe and Zn concentration are listed in Table 4.

**Table 4:** Favorable alleles for grain Fe and Zn concentration.

Trait	Favorable allele	ai *	No. of genotype	Representative genotypes
Fe	PBALC13-1	+10.66	17	L404, L 830, L 4596, L4602, L4603, L4698, DPL21, PL02,P2124, P2125,P2126, P2127, P2130,P3113,P3204,P3208,P15104
	PBALC206-1	+9.73	17	L404, L 830, L 4596, L4602, L4603, L4620, L4648, L4649, L4650,L4698, L5120, L5126, L5253, ILL7663,L7818,L7903
	GLLC563-1	+2.62	20	L404, L 830, L 4596, L4602, L4603, L4620, L4648, L4649, L4650,L4698, L5120, L5126, L5253, ILL7663, DPL15, DPL21, DPL58
	GLLC563-2	+7.72	23	P2102,P2124,P2125,P2126,P2127,P2130,P2205,P2215,P2230,P2233,P2239,P3113,P3204,P3208,P3220,P13104,P13113, P13122,P13135,P13143, P14103,P14201,P15104
	PBALC32-1	+10.58	18	L404, L 830, L 4596, L4602, L4603, L4618, L4620, L4648, L4649, L4650,L4698, LL1231, P2102, P2124,P2125,P2126,P2127,P2130
Zn	PBALC353-3	+8.54	18	P13129,PL129,P2102,P2124,P2126,P2130,P2215,P2230,P2233, P3113,P3208,P13104, P13113, P13143, PL02,L4698,L5253
	PLC62-2	+3.53	10	DPL15,DPL21,DPL58,PL101,PL639,RL1,ILL2581, SKL259,L4590,PL4
	PLC62-4	+9.84	7	P2124,P2126,P2130,P3113,P3204,P3208,P3220

*ai = the phenotypic effect of allelic variation

## Discussion

### Genetic variation in grain Fe and Zn concentration

Breeding micronutrient-rich crops is required to combat micronutrient deficiencies in humans [80]. The characterization of genotypes for micronutrient concentrations in different environments is essential for identifying genotypes rich in Fe and Zn. In the present study, high genetic variation for grain Fe and Zn concentration was recorded. Approximately 29.1% of genotypes had a high mean grain Fe concentration (>70 mg/kg) and 31.2% of genotypes had a low mean grain Fe concentration (<60 mg/kg). Similarly, 22.9% of genotypes had a high mean grain Zn concentration (>50 mg/kg) and 20.8% of the genotypes had a low mean grain Zn concentration (<40 mg/kg). The results indicated that the grain Fe concentration was higher in the indigenous genotypes than in the exotic genotypes. By contrast, the exotic genotypes were relatively richer in grain Zn concentrations than were the indigenous genotypes. The germplasm has been characterized for grain Fe and Zn concentration in wheat [81, 82], rice [83] and chickpea [84, 85]. A wide range of variability in grain Fe and Zn concentration in lentil [86, 87] indicated the role of genotypes and environmental interactions in the expression of these traits. The variation in grain micronutrient concentration of the lentil genotypes at different locations and years in the present study was due to the sensitivity of genotypes toward variations in weather and soil conditions. Soil parameters affect the availability of Zn uptake in plants and control the amount of organic matter and the grain Zn concentration in soil solutions [88]. By hybridizing indigenous Fe-rich grain varieties with exotic Zn-rich grain varieties, the concentration of both micronutrients can be improved simultaneously and the genetic base of indigenous genotypes can be broadened. Biofortified lentil varieties can be sustainable as well as cost-effective for alleviating the micronutrient deficiency, thereby complementing the process of food fortification. Thavarajah *et al*. [89] reported that 100 g of dry lentil can provide the recommended daily allowance of micronutrients (Fe and Zn) in adults. Therefore, lentil can be consumed as a whole grain to meet the daily requirement of grain micronutrient concentration in humans.

Correlation is a crucial aspect of the crop improvement program and is used to improve correlated traits simultaneously or reduce undesirable effects of some traits on another. We observed a positive correlation (r = 0.11) for grain Fe and Zn concentration. Similar results have been reported in other studies [90, 91]. Fe and Zn are found in similar types of foods, and their absorption mechanisms are affected by food compounds. Therefore, the biochemical status of Fe and Zn may be interconnected [92]. A significant correlation between grain Fe and Zn concentration was reported in fieldpea [93], barley [94] wheat [95] and maize [96, 97]. The positive association between these traits suggests some common genetic mechanisms for the uptake, accumulation, and concentration of Fe and Zn. Correlation is very useful in the crop improvement program as a breeder can improve the micronutrient concentration of both the traits (Fe and Zn) simultaneously. Although the levels of Zn and Fe in grains are positively related, fertilization of one element did not affect the grain concentration of the other [98, 99].

### SSR allelic diversity and population structure

SSR markers due to their codominant nature have been widely utilized for genetic diversity, gene tagging, and linkage mapping in numerous crop plants including lentil [31,32,72,100]. In this study, the genetic variability and population structure were analyzed among 96 germplasm with 73 polymorphic SSRs. The mean PIC value was 0.36. Twenty genomic SSRs exhibited higher mean PIC of 0.39 whereas fifty three EST SSRs exhibited mean PIC of 0.35. EST-SSRs located in the proximity of the coding region are associated with the expressed gene/QTL; hence, they are superior markers [101]. The PIC is a critical factor for selecting SSRs for the characterization of germplasm and tagging of genes [102]. PIC offers a more accurate assessment of diversity than does the raw number of alleles because PIC takes into account the relative frequencies of each allele [103]. It also indicates the ability to discriminate among genotypes. Kushwaha *et al*. [104] reported that markers with a PIC value ranging between 0.4 and 0.8 can be considered as informative exhibiting high polymorphism. The PIC observed in the current study were comparable with those reported in previous study by Andeden *et al*. [105], where PIC ranged from 0.13 to 0.79. In our study, the genetic diversity of genomic SSRs was higher than that of EST-SSRs and these findings are consistent with those of previous studies on lentil [100] and barley [106]. A lower level of polymorphism in EST-SSRs may be due to selection against variation in the conserved regions. PIC values of the SSRs PBALC 13, PBALC 206, and GLLC 563 associated with the grain Fe concentrations were 0.33, 0.29, and 0.59, respectively. Similarly, the PIC values of the SSRs PBALC 353, SSR 317–1, PLC 62, and PBALC 217 associated with the grain Zn concentration were 0.34, 0.57, 0.53, and 0.17, respectively.

The population structure explained the presence of six subgroups in the AM panel. (Fig 2). The subgroup SG 5 consisted of 23 genotypes from the Mediterranean region. This group separated the Mediterranean genotypes from the remaining genotypes from India. The initial report [5] indicated that lentil genotypes from India have a narrow genetic base and are genetically more isolated than that of the the ICARDA genotypes. However in the last two decades Mediterranean material has been used for broadening the genetic base. Previous studies on lentil diversity by using molecular markers have revealed mostly two distinct groups, namely South Asian and all other origins [86,107,108] results from PCoA and cluster analyses also demonstrated a narrower genetic variability among the Indian material. Similarly Mekonnen *et al*. [109] and Koul *et al*. [110] reported five subgroups in the lentil germplasm. The genotyping of available genetic diversity has demonstrated the need for incorporation of the exotic germplasm into breeding programs for broadening the genetic base.

### Association analysis and favorable allele identification

In the present study, we identified markers that are consistent across environments. Three SSRs were associated with the grain Fe concentration and four SSRs with the grain Zn concentration. After the validation of these trait-associated SSRs, the markers can be used for identifying genes/QTLs regulating grain Fe and Zn concentration. Furthermore, they can be useful in the marker-assisted genetic enhancement programs. The markers exhibiting an association in more than two environments were considered more reliable than the markers present in a particular environment. The SSRs PBALC 13 and GLLC 563 accounted for phenotypic variation of 11%, whereas PBALC 206 explained 9% of phenotypic variation for the grain Fe concentration. For the grain Zn concentration, PBALC 353 exhibited the highest phenotypic variation (21%), followed by SSR 317–1 (18%), PBALC 217 (16%), and PLC62 (14%). Similar level of trait variation for grain Fe and Zn concentration was recorded in fieldpea [93] and chickpea [84]. The size of our lentil germplasm panel was adequate. Even with small population size the high quality extensive phenotyping offers a reasonable basis for the association of mapping studies [111]. Association mapping studies in bean [112], peanut [113], barley [114], tomato [115] and sugarcane [111] also used approximately 90 genotypes. Atwell *et al*. [63] reported that for some of the traits, significant results can be achieved in a population size of less than 100. The putative functions of PBALC 217 reported by Kaur *et al*. [51] were assigned through comparison with the non redundant sequence database at the National Center for Biotechnology Information by using the basic local alignment search tool (BLAST) BLASTX program (http://blast.ncbi.nlm.nih.gov/Blast.cgi). This SSR exhibited homology with dormancy or auxin-associated protein in *Medicago*. *truncatula* (E value = 3e-42). Similarly, Upadhayay *et al*. [85] reported the auxin/IAA as a known/putative function of SNP (CakSNP1628/SNP55) associated with the seed Fe concentration.

By using genomic SSRs, association mapping has been successfully demonstrated in rice [116], wheat [117] and chickpea [118]. Our study in lentil using SSRs is the first attempt to identify QTLs or genes for grain Fe and Zn concentration. Gupta *et al*. [119] used 50 SSRs for the mapping QTLs for agronomic traits in foxtail millet. Gorafi *et al*. [82] used 70 SSRs for the identification of linked markers for grain Fe and Zn concentration in wheat. Lou *et al*. [120] used 90 SSRs for the mapping QTLs for agronomic traits in Fescue. Gyawali *et al*. [121] used 84 SSRs for association mapping in *Brassica napus*.

The association mapping approach was useful in identification of marker loci linked with the grain Fe and Zn concentration. This approach also aided in mining alleles and further utilizing these favorable alleles with the maximum positive effect in marker-assisted selection, as suggested by Wan [122]. The identified favorable alleles can be used in the lentil breeding program for improving grain Fe and Zn concentration. For the grain Fe concentration, the maximum positive effect for the favorable allele PBALC 13–1 was recorded in seventeen genotypes (L 404, L 830, L 4596, L 4602, L 4603, L 4698, DPL 21, PL 02, P 2124, P 2125, P 2126, P 2127, P 2130, P 3113, P 3204, P 3208, and P 15104). Similarly, for the grain Zn concentration, the maximum positive effect for the favorable allele PLC 62–4 was recorded in seven genotypes (P 2124, P 2126, P 2130, P 3113, P 3204, P 3208, and P 3220) originating from ICARDA. These genotypes can be utilized in a hybridization program for simultaneous improvement of grain Fe- and Zn-rich varieties. Similar studies have also been performed in other crops such, wheat [106] and tomato [115].

Biofortification (breeding micronutrient-rich crops) of lentil can be achieved through plant breeding without affecting the yield or quality. The process like transfer of other traits is tedious and involves screening of germplasm, hybridization, study of mode of inheritance, molecular marker-assisted selection, and regular phenotyping of segregating populations. The approach is a sustainable and cost-effective solution for delivering micronutrients [123] and has emerged as an agriculture-based strategy to fulfill the nutritional requirement of malnourished people throughout the world. Considerable knowledge has been obtained on the molecular mechanisms affecting the accumulation of Fe [124] and Zn [125] in plants. In the future, these studies can be applied to develop crops with enhanced mineral concentration with the help of biotechnological tools in conventional breeding. In this study, markers were identified as being linked to grain Fe and Zn concentration. These identified SSRs can be further validated and deployed in marker-assisted selection for developing grain Fe and Zn rich lentil varieties. Superior positive alleles can be pyramided by hybridization for enhancement of grain Fe and Zn concentration.

## Supporting information

S1 Fig Histogram exhibiting distribution of genotypes for grain Fe and Zn concentration. (TIF)

S2 Fig Quantile–quantile plots to study the association of 73 SSRs markers with the grain Fe and Zn concentration in five datasets. (TIF)

S1 Table Data for grain Fe and Zn concentration (in mg/kg seed) across locations/years. (XLS)

S2 Table Genotypic and phenotypic correlations matrix for grain Fe and Zn concentration in lentil. (XLSX)

S3 Table Soil analysis data of different locations. (XLSX)
