# Supplementary figures and images for: Association mapping unveils favorable alleles for grain iron and zinc concentrations in lentil (Lens culinaris subsp. culinaris)
Source: PLoS One. 2017 Nov 21;12(11):e0188296. doi: 10.1371/journal.pone.0188296 (PMC5697819; doi:10.1371/journal.pone.0188296)

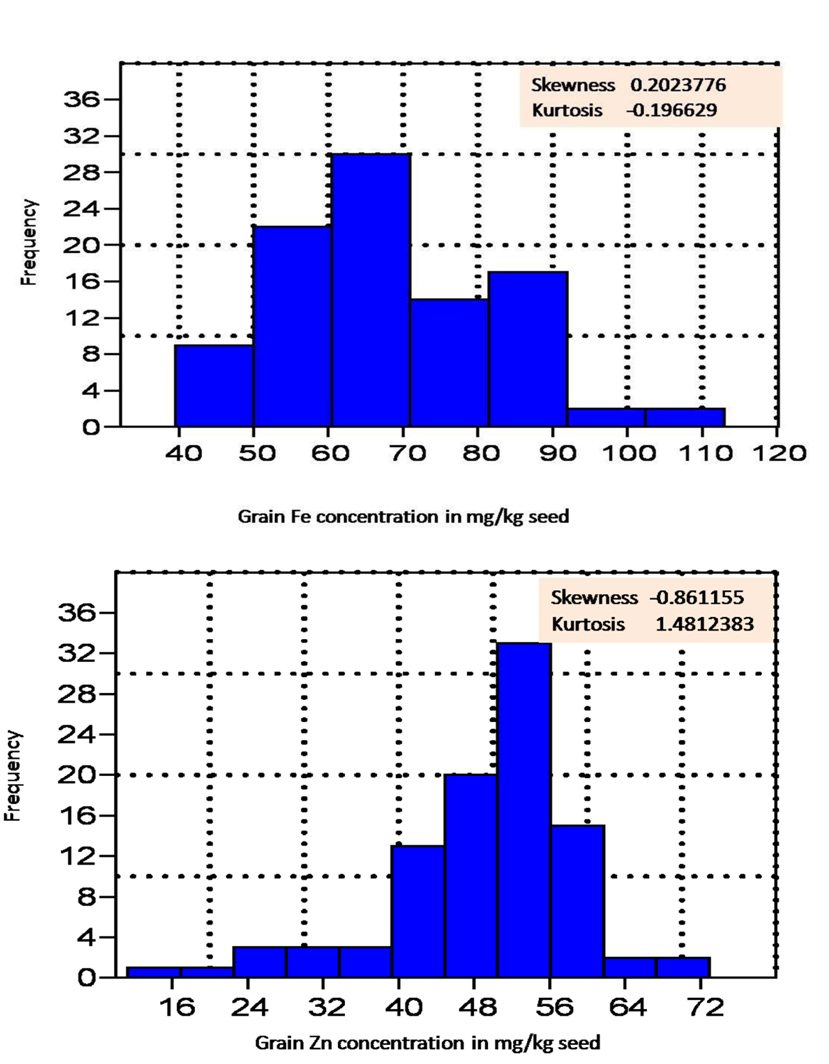

Supplement: S1 Fig — (TIF) [file pone.0188296.s001.tif]

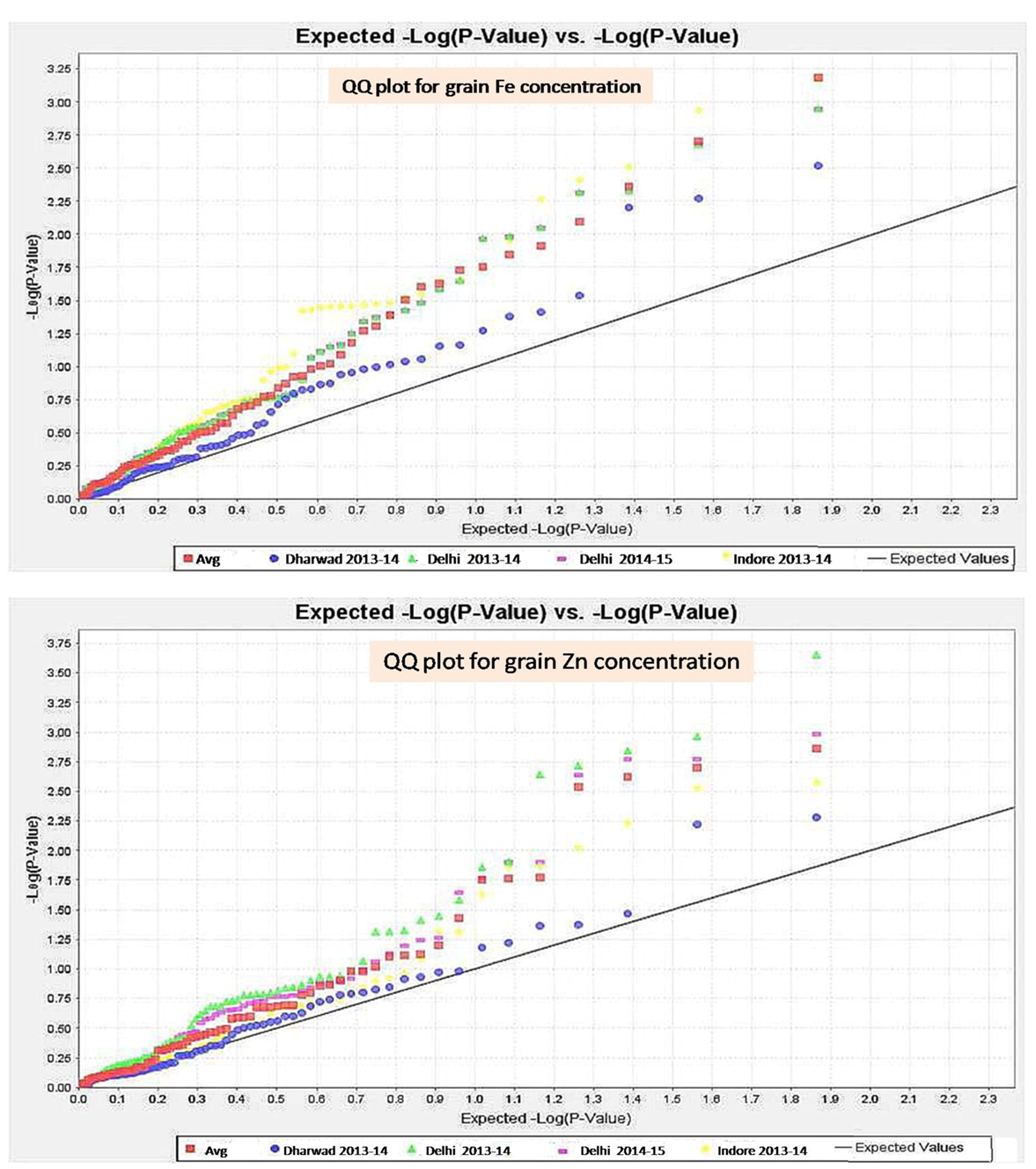

Supplement: S2 Fig — (TIF) [file pone.0188296.s002.tif]
